# ﻿A survey of keys for the identification of newly described insect genera: recommendations for authors, reviewers, editors, and publishers

**DOI:** 10.3897/zookeys.1215.130416

**Published:** 2024-10-11

**Authors:** Laurence Packer

**Affiliations:** 1 Department of Biology, York University, 4700 Keele St., Toronto, ON M3J 1P3, Canada York University Toronto Canada

**Keywords:** Best practices, biodiversity assessment, ease-of-use, entomology, identification keys, images, key construction guidelines, taxonomy

## Abstract

Large numbers of new taxa are described annually and while there is a great need to make them identifiable, there seems little consistency in how this might be facilitated. 427 papers published in 2021 and 2022 were surveyed, which described 587 new insect genera. Only 136 of these papers included keys, and these allowed the identification of 233 of the new genera (31.9% of papers and 39.7% of the new genera). The proportion of papers that included a key varied significantly among insect orders but not among the handful of journals wherein the bulk of the new genera were described. Overall, for 17 key-related variables assessed in a binary fashion (optimal vs suboptimal), the average key had almost six criteria that were scored as being suboptimal. For example, less than one-fifth facilitated retracing and less than 12% had illustrated keys where the images were conveniently located close to the relevant key couplets. Progress towards confirming a putative identification was possible in all papers, through the inclusion of a diagnosis, habitus images, or both.

Based upon this analysis, and expanding on previous suggestions for key construction, 23 recommendations are made on how to make an identification key maximally useful for users and I indicate the relative ease with which each could be adhered to. Identification keys should accompany all new taxon descriptions, guidelines for effective key construction should be added to journals’ instructions to authors, editors and reviewers should check keys carefully, and publishers should be attentive to the needs of users through, for example, permitting duplication of images to make keys easier to use. Recommendations are likely relevant to all levels in the taxonomic hierarchy for all organisms, despite the data being derived from generic-level keys for insects.

“… the most important impact of taxonomy is the usage of identification keys”

(Krell 2002)

## ﻿Introduction

With increasing concern about declines in abundance and diversity of insects (e.g., Didham et al. 2020; Wagner 2020; Sánchez-Bayo and Wyckhuys 2021), there is a growing need for efficient tools to identify them. Yet resources to permit insect identifications are scattered and diverse, vary greatly in how easy they are to use and the extent to which they are taxonomically and/or geographically relevant. Furthermore, new taxa are being described at a high rate, but despite the recommendations of Walter and Winterton (2007), there seem to be no consistently applied criteria as to how they are to be made readily identifiable by others.

There are four means whereby new taxa can be made identifiable by those other than the original author(s): the diagnosis, the description, illustrations, and an identification key. In this paper I assess the current status of the identification keys (if provided) that accompany the description of new insect genera. I also assess the frequency with which the keys referred to appropriate illustrations and whether diagnoses were also provided (a formal assessment of the nature of insect genus level diagnoses is in preparation). Accurate assessment of the quality of descriptions for a wide range of insect taxa is likely beyond the ability of any one entomologist and I also do not assess the validity of the new genera as this would require a deep understanding of the systematics of most of the world’s known biota. However, it is worth noting that a small minority of new genus descriptions were associated with the phylogenetic information that would provide a thorough justification (pers. obs.).

Generic level keys were chosen for assessment because their number was expected to be tractable for analyses for all insect orders for which new genera were described and thus the results of this analysis should be broadly applicable to entomological taxonomic research. Furthermore, at publication, the information in the paper describing a new genus would likely be the only way it could be identified. Consequently, I assessed papers for newly described insect genera published over two years starting from January 2021 to see whether they included an identification key and if so, how well the key fit a range of criteria (see below).

## ﻿Methods

I searched for relevant papers using Scopus searches for “new gen*” or “gen* n*” for all insect orders (including names that are not generally in current use such as Heteroptera, Homoptera, and Isoptera), during the time period January 2021 to December 2022. The journal Entomological Review publishes translations of papers originally in Russian wherein taxonomic acts often formally date to the year previous. Those discovered in English translation in the above two years were included in the sample. Papers were downloaded using Google Scholar, ResearchGate, or the library resources available to me at York University. I requested PDFs from corresponding authors of papers in Zootaxa (wherein the largest number of new insect genera were described) when these were not otherwise available. I separated out those papers that included one or more identification keys for analysis. Those that did not include a key were not investigated further except for calculation of the proportion of papers that included a key and the proportion of new genera that were associated with an identification key. This was calculated separately for each insect order and journal as well as in aggregate.

Five journals had far more new genus descriptions than others and in the text below they are denoted by the following abbreviations: **EJT** – European Journal of Taxonomy, **SE** – Systematic Entomology, ZJLS – Zoological Journal of the Linnean Society, **ZK** - ZooKeys, **ZT** – Zootaxa.

I did not include papers that dealt solely with fossil taxa because of the difference in approaches required with the study of such material. Similarly, I only included keys to adult insects unless adults were not treated in the paper as is commonly the case with some groups (Ephemeroptera, Aleyrodidae, male Strepsiptera).

Some keys treating new genera also included fossils and/or taxa above or below the level of genus (e.g., tribes, genus groups, subgenera, species) or included couplets that led to groups that were not further treated in the key. In such cases, only couplets that eventually led to a generic level identification were included in numerical analyses. However, if a couplet that led to a different taxonomic level in one lead, but to further couplets leading to genera in the other, these were included among the statistics with respect to both the number of couplets required to get to a generic level identification and the proportion of leads that were illustrated. As a result of these issues, the number of couplets assessed and included in tabulations sometimes differed from those in the published key.

Some papers provided more than one genus-level key either because males and females were treated separately (*n* = 3 papers, Cumming et al. 2021; Cumming and Le Tirant 2022; Benda et al. 2022) or because the new taxa belonged to different intermediate taxonomic levels (*n* = 2 papers, Brunke 2021, Sharkey et al. 2021). Thus, the number of keys assessed was slightly larger than the number of papers that included keys.

**Figure 1.**
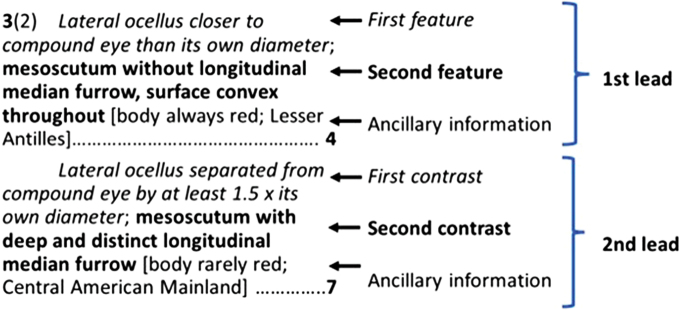
Schematic of a key couplet to outline some terms used in this paper.

Terms associated with key structure are shown in Fig. 1. This is a hypothetical example couplet for a dichotomous key where features are treated in parallel. Most, but not all, keys followed this approach (see below). Each half of a dichotomous couplet is termed a lead and when more than one discriminating aspect of the organisms is included, they are henceforth called features (note that Walter and Winterton (2007), among others, use the terms character and character state for the same key components, but it seems desirable to avoid those words due to their usage in phylogenetics). It is common for authors to add ancillary information that might not be perfectly discriminatory of the groups defined by the two leads. Such information may be helpful and is usually shown in brackets before the next couplet number or the identification. Ancillary information commonly includes geographic features as in the example given below. I did not assess ancillary information as it is, by design, not decisive.

For each of the keys, I assessed the variables outlined below. Initially, I used the criteria noted by Walter and Winterton (2007: table 2) but it rapidly became evident that there were additional issues that required consideration. When the same, or a similar, topic was treated by them the fact is denoted by WW# where # relates to the number in their table 2.

1. Was the text freely available and was the key in the main body of the paper or as supplementary materials only? This was assessed at two levels of availability: a) generally freely available through Google Scholar searches for open access papers among those included in the sample and, if not, b) whether the paper was freely available through subscription content available through York University Libraries [this involves a system called Omni, which is the discovery service used by a large proportion of universities in Ontario]. I note that institutions using different discovery services would likely have access to a different suite of journals.
2. Was it possible to determine that the paper included a key by using standard online search protocols? This was done by checking whether the term “key” was found in the title, abstract, or keywords in each paper.
3. Was it possible for the user to ensure that the key was relevant to the specimen to be identified by stating the discriminating features of the taxonomic group and sex (and for social insects, caste) to which the key applied? These variables were assessed by searching the paper for descriptive or diagnostic information for the taxon at the level to which the key as a whole applied and whether it was overtly stated that the key applied only to one sex or, where relevant, to one caste.
4. For the variables assessed in iii), was it easy to locate the necessary information? Where was it provided in relation to the key?
5. Is the key of the standard dichotomous structure and free of errors or surprises? A few keys were of the yoked style, wherein all taxa agreeing with the first lead of the first “couplet” are identified before the second lead of that “couplet” is reached. Errors arise through incorrect numbering and surprises may be encountered through the insertion of a non-dichotomous “couplet” among typical dichotomous ones. An additional non-standard key component is the leap-frog couplet, of which the following would be an example:

3(2) Head longer than wide; hind tibia with apicoventral spines 9

– Head shorter than wide; hind tibia lacking apicoventral spines, surface flat 4

In this instance, users may go to couplet 4 instead of couplet 9 from the first lead simply because it is expected that the first lead should take the user to the next couplet as this is standard practice.

1. Does the key allow retracing? (WW7). The standard way of denoting this is shown in Fig. 1 and the above example; in each case, couplet three was reached from couplet 2. This is useful because users often reach a dead-end where the specimen seemingly agrees with neither lead and going backwards unassisted or restarting the key from the beginning is time-consuming and increasingly so the longer the key.
2. How many steps does it take to get to an identification? (WW8). This variable requires some explanation and concerns the extent to which keys are fan- or comb-shaped and is an important characteristic for a user as the average number of steps to attain an identification increases with an increase in number of taxa far faster in comb-shaped keys than in fan-shaped ones (Walter and Winterton 2007; Packer et al. 2016). For example, the average number of steps from the beginning to identify the taxa in a 16-taxon comb-shaped key is 8.4 but varies from one to 15; whereas for a perfectly fan-shaped key for the same number of taxa it is only, and always, 4. For 128 taxa, these averages increase to 64.5 in a comb-shaped key but only to seven for a fan-shaped one (see the discussion for some added complexity concerning the number of steps to achieve an identification).

I calculated the average number of couplets leading to all the genera in a key averaging in cases where the same taxon came out more than once. I also assessed where those numbers fell between the calculated maximum and minimum numbers of steps that could have been required. For these calculations, I used the equations below.

The average maximum number of steps per taxon, i.e., for an entirely comb-shaped key, for n taxa is:

S_MAX_ = ((n+1)/2) -1/n

whereas for a maximally fan-shaped key the average number of steps required for identification is:

S_MIN_ = (Q*(2^Q^ - x) + (Q+1)*2x)/n

where Q = Ꙇlog2(n)ꓙ and x = n - 2Q.

(surrounding an expression by Ꙇ and ꓙ indicates that its numerical value is to be rounded down to the next lowest integer, e.g., both 4.01 and 4.99 become 4).

Note that this is the same as the equation given in Packer et al. (2016), i.e., log(n)/log(2), only in the special case where n belongs to the geometric series base 2, i.e., when x = 0.

1. Are couplets monothetic? (WW2) A monothetic couplet is one in which only a single feature is used in the two leads. Such couplets will be impossible to use if that structure is missing and unnecessarily difficult to use if the structure is obscured in the specimen at hand or requires dissection. I counted the number of couplets in each key that were monothetic.
2. Are the features in a couplet dealt with in parallel in both leads (WW4) and is each contrasted? If the sequence of observations in the first lead is the same as in the second lead, it is easier to make the necessary comparisons than if they are not. If one feature is not contrasted, then that comparison is impossible and inclusion of that feature not only unhelpful but wastes users’ time. I compared the leads for each couplet to detect non-parallel sequences or absent contrasts.
3. Аre the features ambiguous? (WW3). Another way of stating this is to ask whether a couplet might be indecisive. There are multiple ways in which a couplet might be indecisive. Terms such as “usually”, “often”, “normal”, etc., will not work if the specimen at hand is not of the “usual” phenotype.

Another way in which a couplet might be indecisive would be if a non-overlapping range of variables was given. For example, body length 9–12 mm versus body length 11–15 mm.

Similarly, features that are relative may be difficult for a user to interpret. For example, stating that a structure is strongly or weakly convex is a comparison that requires elaboration; the author of a key will know what is strongly convex for the taxonomic group under study but what is “strongly” convex in that group may be “weakly” convex in another. I noted all instances where a couplet was partially, or entirely, made up of relative statements and whether the difficulties were resolved through reference to illustrations. Optimally, the taxon with the least convex “strongly convex” condition and the taxon with the most convex “weakly convex” condition should be illustrated. It was not possible to evaluate the extent to which this approach was taken in the illustrated exemplars, but I considered a potentially indecisive feature to be resolved if both leads were illustrated for the relevant feature(s).

I noted all instances where a couplet contained indecisive features or whether entire couplets were ambiguous.

1. Is at least one feature from each lead illustrated (WW5) and if so, are the images close to the relevant key couplet? (WW6). I assessed the proportion of leads that cited at least one figure and where the images were in relation to the relevant couplets.
2. Are diagnoses provided for the genera? (WW9). Diagnoses are meant to be relatively short statements that note the characteristics of the named taxon either as a list or a combination in a manner that permits its differentiation from similar taxa (Austral Entomology 2024; see also Borkent 2021; Rheindt et al. 2023). A user may go to the diagnosis for a taxon as a confirmatory step, once a putative identification has been made using the key (Borkent 2021). I checked whether confirmation of an identification was possible through presentation of diagnoses for the new genera or all genera in the key.
3. Are habitus images provided? With the increasing availability and affordability of good quality imaging, provision of a picture of the organism could relatively easily allow the user to decide whether the putative identification might be correct. I assessed whether papers had habitus illustrations of new genera and whether they were photographs, drawings, or scanning electron micrographs.

Some keys could not be assessed for all variables considered. For example, keys that were emendations of previously published ones could not always be assessed for the number of couplets taken to get to an identification of the new genus (or any other) because that information was presented elsewhere, was not always accessible and I wanted to assess the articles as stand-alone papers. However, data for most of the other variables noted above could be assessed for just the emendations and one paper (Engel et al. 2022) emended the beginning of a key and so it could be included in all analyses relevant to just the new genus [Anderson and Bermudez-Higinio (2022) emended two previous keys using a single couplet that would fit at the beginning of the key for one, and much later in the key in the other; it was included as the latter]. Similarly, keys that included trichotomies were not assessed for minimum and maximum numbers of couplets required to get to an identification because equations 1 and 2 rely on all couplets being dichotomous. They could, however, be included for most of the other variables. As a result of these considerations, sample sizes vary among different analyses.

Taking the above criteria ‘en masse’, it is possible to assess the extent to which keys are suboptimal. To achieve this, the following variables were assessed as binary: whether 1) the key was available, 2) the term key would be located in standard key word searches, 3) the characteristics a specimen at hand must possess for the key to be relevant to it were overtly stated, 4) it was stated clearly that the key applied to just one caste, sex, and lacked sex specific features or couplets based on sexual dimorphism, 5) there were no formatting errors, 6) there were no leap-frog couplets, 7) all couplets were dichotomous, 8) retracing was possible, 9) the key was as fan-shaped as possible, 10) there were no monothetic couplets, 11) all features were contrasted, 12) no couplets were entirely indecisive, 13) images were available for each lead and 14) conveniently placed close to the couplet, 15) all taxa were diagnosed, 16) a habitus image was provided, 17) the key was an emendation but did not require the previous key to be usable. Thus, each key was assessed for an overall score out of 17. However, because some criteria were not applicable to some keys (e.g., whether retracing was made possible is not relevant to a key with a single couplet), overall key scores were also assessed through the percentage of relevant criteria for which they were suboptimal.

## ﻿Results

### ﻿Summary data

I found 427 papers that described a total of 587 new genera although 28 papers, describing 34 new genera, were not available for a range of reasons: unavailable through university library resources or Google Scholar combined with a lack of response to requests for a pdf or inactive or unlocatable corresponding author email addresses; an additional paper, describing one genus, was not written in English. Thus, the sample analysed included a total of 398 papers that described a total of 552 new genera. Only 136 papers (34.2%) included identification keys that treated 232 (42.0%) of the new genera (all papers are listed in Suppl. material 1 and the number of new genera in each is shown in Suppl. material 2, column H). These were among the total 1507 genera included among the keys that incorporated a total of 1448 couplets (note that the total number of couplets should equal the number of genera in all keys minus the number of keys; however. this assumes all genera came out just once in each key and that all keys were fully dichotomous, neither assumption was upheld – see below).

Except in cases where only a few papers illustrate a point being made, consultation of Suppl. material 2 is required to find out which papers exhibited the state for a particular variable.

A total of 141 keys were found among the 136 papers (some papers included separate keys for males and females or to different taxonomic subgroups, see Suppl. material 2, column J). Ten keys were emendations to earlier ones (Suppl. material 2, column K) of which only one (Engel et al. 2022) did not require the original key to be available to permit identification of the new genus. Four were to incomplete and seemingly unidentifiable subgroups of genera (“to the species described in this paper” Namaki-Khameneh et al. 2021a; “selected genera” Sublett and Cook 2021; “to the genera discussed” Yasunaga et al. 2021; “most of the genera” He et al. 2021). Two other papers provided keys to a “phyletic group” (Polak and Mulaomerović 2021) or “phyletic series” (Ćurčić et al. 2021); the reference for both terms is Jeannel (1924), a 436-page article in French that seems difficult to access.

Among the six orders that were represented by more than 20 papers (see Suppl. material 2, column D), there was significant variation in the proportion of papers that included a key (χ^2^ = 36.6, p = 7.19 × 10e^-7^), ranging from 5.3% for Lepidoptera to 47.1% for Coleoptera. The proportion of papers with a key among the remaining orders combined (maximum number of papers *n* = 8) was 44.4% not significantly different from the papers to the six more frequently studied orders combined (χ^2^ = 2.04, p > 0.1).

Among the six journals with more than ten papers describing new genera (see Suppl. material 2, column C), the proportion of papers that included a key ranged from 33.6% for ZT to 50.0% for EJT and ZK. Variation among journals in the preponderance of papers with keys was not significant (χ^2^ = 6.13, p = 0.29). The remaining journals had eight or fewer papers describing new genera but the proportion among their total that included a key was only 25.5%, significantly less than among the above six journals combined (χ^2^ = 7.95, p < 0.005).

**Figure 2.**
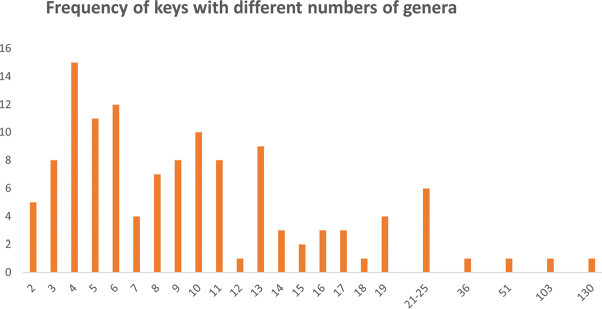
The frequency of keys that were to different numbers of genera. Note the disjunctions in the y-axis due to the small number of keys with more than 19 genera.

The number of genera in keys ranged from two to 130 with an average of 11.43 but with far from a normal distribution (Fig. 2; see also Suppl. material 2, column I).

The number of couplets in a key was not always what might be expected - the expectation being n-1, where n is the number of genera treated in the key (Suppl. material 2, column H). This was for a range of reasons, primarily when the same genus came out multiple times in the key (e.g., Viswayjothi and Clark 2022). The reasons are explained in the footnote to the relevant column.

Twenty-one keys (14.9%) were to the genera within a family, 35 (24.8%) were to those within a subfamily, 50 (35.5%) treated genera within a tribe, 11 (7.8%) were to those of a subtribe, 21 (14.9%) were to groups of genera and three (2.1%) were to ecologically defined subsets of higher-level taxa (Suppl. material 2, Column D).

Almost half of the keys (45.39%) were global in extent for the higher-level grouping to which the new genus belonged, although often the entire group was geographically restricted. Forty-nine keys (34.75%) were for a continental fauna or one that spanned two adjacent continents. Twenty-six (18.4%) were to single countries (data for Australian taxa were included in the continent statistics) or for a few nearby countries (e.g., India / Sri Lanka and Bosnia and environs) and two (1.4%) were to a part of a single country (see Suppl. material 2, column E).

### ﻿Key assessment

#### ﻿i) Was the text freely available?

Of the 136 key-containing papers, 101 (74.3%) were freely available through Google Scholar, ResearchGate or open-source journal websites. Another seven (5.1%) were available through library resources (most of the previous 101 were also available through this route). Thirty (20.6%) were not available to me from either of the aforementioned approaches but were received after requesting a pdf from the senior author. This method of obtaining the papers permitted the identification of 50 new genera (21.5%) and 279 genera (18.9%) in total. When these are added to the 29 papers for 35 new genera for which citations were found but the papers seemingly unavailable (see above), a total of 59 papers (13.8%) for 85 new genera (14.5%) were either not accessible at all or required contacting the author.

Two of the keys were available only in the supplementary materials (Castro-Huertas et al. 2021; Xu et al. 2021), relevant to seven new genera (3%) and 154 genera (10.2%) in total.

#### ﻿ii) Was the term “key” discoverable using standard online search protocols?

The term “key” was first found in the title of twelve papers (8.8%), among the keywords in seven (5.2%), and in the abstract for 77 (56.6%). Thus, the fact that the paper included a key would be readily discovered using standard search terms for 96 (70.6%) of the papers, representing 169 new genera (72.5%) and 1009 (70.2%) of all genera (Suppl. material 2, column L).

#### ﻿iii) Was it possible for the user to ensure that the specimen to be identified belonged to the group for which the key was constructed through the text stating the characteristics that had to be present for the key to be useful and/or sex or caste of the specimen?

Twenty-one papers had keys to genera within a family for which instructions on how to identify the group might be considered unnecessary, nonetheless, two papers (Ardila-Camacho et al. 2021; López-Pérez, Zaragoza-Caballero 2021) stated how to do so. Fifty-nine keys (41.8%) were in papers that explained how to identify the taxonomic group below the family level for which the key was relevant. Five of these stated the apomorphies that helped define the group. Seven papers stated a single feature, though in two of these (Huo et al. 2022; Yin and Kurbatov 2022) the feature required males to be available and so use of the key might not be embarked upon for female specimens. Nineteen papers provided a formal diagnosis of the group keyed (most, but not all, when a new taxon above the generic level was also being described). An additional 28 keys were associated with lists or combinations of features that users might check to determine whether the key was relevant to a specimen at hand but without a formal diagnosis. The number of features stated to make the key relevant ranged from one to 40 and averaged 8.2 (but with high variance: SD = 9.5; Suppl. material 2, column N). Thus, overall, 53.7% of all genera and 52% of the new genera were in keys where how to determine whether the key might be appropriate for a specimen was not explained (below family level).

For twelve keys (8.5%) for a total of 14 new genera (6.0%), both sexes were known but the key permitted identification of only one without overtly stating so (Suppl. material 2, column O). This issue arose for 181 (8.2%) of all genera.

Five keys were to specific castes of social insects, three to ant workers, one to bee workers, and one to termite soldiers. In one key to worker ants (Camacho et al. 2022) and one to bees (Engel et al. 2022) (1.4% of all keys), for one new genus each, it was not clearly stated in the heading to the key that it applied only to one caste (Suppl. material 2, column O). This issue was relevant to 13 genera in total (0.9%) and two new genera (0.9%).

#### ﻿iv) Where was this information placed within the paper?

The placement of the information enabling the user to decide whether the key was taxonomically appropriate was not consistent. Most usefully, it was immediately above the key, as in 23 cases (37.7%). It was in the introduction section of the paper also in 23 articles, elsewhere in the results in 12, and once each immediately beneath the key, in the methods or the diagnosis for the new genus.

#### ﻿v) Is the key of the standard dichotomous structure without errors or surprises?

Reasons for there being a different number of couplets in a key than expected based upon the number of genera are explained in Suppl. material 2, column H. In eleven keys, one or more genera came out more than once. In most cases only one or two genera keyed out twice but in Viswayjothi and Clark (2022) 37 of the 130 genera came out between two and five times although only two of the ten new genera were repeated, each twice. Two keys (Huo and Du 2021; Nieves-Aldrey 2022) had more than one genus coming out in one lead so those were not separable, though the newly described genera were not among them.

Some keys did not have the standard dichotomous structure: six had one or more trichotomies or a quadrichotomy, one had a “looped” key structure whereby the same couplet could be reached through two different paths (Tshernyshev 2021) and three were in the yoked style wherein all taxa agreeing with the first half of the first “couplet” were keyed before the second half of that “couplet” was reached (Storozhenko 2021; Davidian 2022; Kluge et al. 2022) (Suppl. material 2, column Q). None of these issues inevitably add to the potential for the key failing, though they may increase the probability of user error.

Four keys had errors in numbering with duplicated and/or missing and/or incorrect couplet numbers (Castro-Huertas et al. 2021; Jałoszyński 2021; Lucañas 2021; Polak and Mulaomerović 2021) and a fifth (Namaki-Khameneh et al. 2021b) lacked numbers at the beginning of any couplet (Suppl. material 2, column R). I found no errata corrections associated with errors in these papers (search conducted in May 2024), although Jałoszyński (2021) has been superseded by Jałoszyński (2024) which contains a key to the world genera of the relevant group. Thus, five keys were unnecessarily difficult to follow due to errors in formatting and these difficulties applied directly to 37 (2.5%) genera in total, only three of which (1.3%) were newly described (these numbers are less than the total number of genera and new genera in these papers because only those taxa directly affected by the numerical errors were included in the calculations).

Leap-frogging was found in five keys 4.2% of those for which it would be possible (i.e., keys that had both three couplets or more and that were in a standard dichotomous format; Suppl. material 2, column S). In most cases a single couplet suffered from this, though in Gaimari and Havill (2021) all couplets where neither lead resulted directly in an identification had the leap-frog sequence. Overall, 58 genera (4.4%) were affected by leapfrogging, and six of them were new (2.6%).

#### ﻿vi) Does the key allow retracing?

Of the 120 keys for which retracing may have been sensible (i.e., those with more than two couplets and without the yoked format) only 19 stated which couplet led to the current one, while one more provided that information for the sole couplet that did not continue from the one immediately above it (Prathapan and Konstantinov 2021). Thus only 20 keys (16.7%) where retracing might have been sensible permitted it (Suppl. material 2, column T). This applied to 22.6% of both all genera and new genera. While retracing steps may be deemed unnecessary in keys with relatively few couplets, the number of couplets in keys with retracing versus those without it was not significantly different (z = 1.3232, p = 0.19) and the shortest keys with retracing had three couplets, the longest one without it had 186.

#### ﻿vii) How many steps does it take to get to an identification? (WW8).

**Figure 3.**
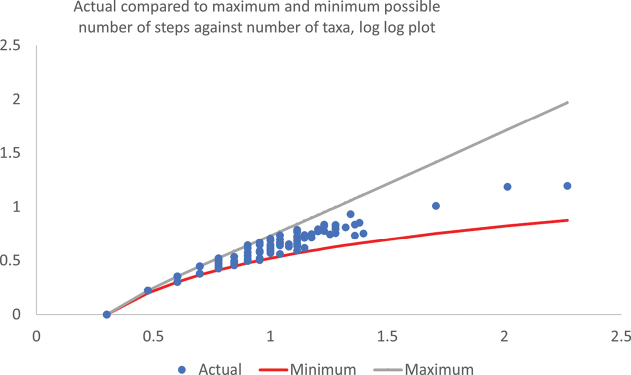
Log-log plot of mean number of steps required to obtain an identification against number of taxa in the key (blue dots) compared to the minimum for a fan-shaped (red line) and maximum number of steps (grey line) for a comb-shaped key.

The actual average number of couplets leading to all the genera in a key is provided in Suppl. material 2 column U. The same data, as well as the maximum (for a comb-shaped key) and minimum (for a maximally fan-shaped key) using equations 1 and 2 (see methods), are shown on logarithmic scales, in Fig. 3.

Thirteen keys (11.1% of those to more than three genera) were as fan shaped as possible given the number of taxa in the key (Suppl. material 2, column V). These keys involved a total of 56 genera, 19 of which were new (4.4% and 2.3% of the totals respectively). Unsurprisingly, these were all to relatively few taxa (with an average of 5.3 genera, the largest key that was as fan shaped as possible was to 9 genera), significantly fewer genera than in keys that were not perfectly fan-shaped (z = 3.93, p < 0.0001).

#### ﻿vii) Are couplets monothetic?

Nine keys (6.4%) relevant to one new genus each (3.4%) were composed of only monothetic couplets, whereas 38 (27.0%) had no couplets based on a single structure (Suppl. material 2, column W). Overall, the identification of 1212 (77.1%) genera required negotiation of one or more monothetic couplets, 167 of these genera were new (71.7% of all new genera). The identification of all genera in a key was subject to monothetic couplets in 69 cases (48.9%), most often due to the first couplet being monothetic. The average proportion of couplets that were monothetic for one or both sexes was 33.9% on a per paper basis and 38.5% across all 1445 couplets. The proportion of couplets that were monothetic per key is shown in Suppl. material 2, column X.

#### ﻿viii) Are the features in a couplet dealt with in parallel in both leads and contrasted?

I found only two exceptions to sequences of features being the same in both leads in a couplet (Allsopp 2022; Kirejtshuk and Kovalev 2022), although identification of the new genus was not dependent upon such a couplet in the first of these. I found nineteen keys where a feature in one lead was not contrasted in the other (Suppl. material 2, column Y), but no single couplet in these keys was entirely composed of uncontrasted features except in one emendation key for a single new genus (Gilasian et al. 2021).

#### ﻿ix) Are features included in a couplet ambiguous or indecisive?

Such features were found in 89 keys (63.1%) and made up all parts of one or more leads in 30 (21.3%) although in three of these (Storozhenko 2021; Zettel and Laciny 2021; Pacheco et al. 2022) the ambiguities were resolved through illustration. Thus, 27 keys (19.1%) had couplets sufficiently indecisive as to make identification of some taxa in the key uncertain (Suppl. material 2, column Z). This uncertainty applied to 48 of the 233 new genera (20.6%).

#### ﻿x) Are features illustrated and if so, are the figures close to the key couplet to which they are relevant?

Sixty-three keys (44.7%) had no figures cited in any couplet encompassing 105 new genera (45.1%) and 653 genera overall (43.3%). At the opposite extreme 28 (19.9%) had at least one figure for each lead, relevant to 40 (17.2%) of the new genera and 239 of all genera (15.8%) (Suppl. material 2, column AA). Overall, the average proportion of leads that were illustrated was 33.8%.

With respect only to those couplets leading to newly described genera, 27.2% of keys had one or more features illustrated in each lead, an additional 9% had only the half of the couplet leading to the new genus illustrated, and 3.6% illustrated only the alternative lead (i.e., the one not leading to the new genus).

Seven keys had the images right next to the couplets and eight had them on an adjacent page (Suppl. material 2, column AB). Two keys had either one (David et al. 2021, the one most relevant for the new genus) or all (Colombo et al. 2022) images hyperlinked to the couplet. Other figures cited in keys were dispersed throughout the manuscript except in three papers (Ślipiński et al. 2021; Yeshwanth and Konstantinov 2021; Viswayjothi and Clark 2022) in which they were grouped, along with all other figures, at the end of the paper.

#### ﻿xi) Are diagnoses provided for the genera?

Eight keys (5.7%) had no diagnosis even for the new genus/genera being described (11 new genera, 4.7%) and another 13 (9.2%) (for 15 new genera, 6.4%) had no diagnosis section but had the kind of information that might be expected in one somewhere in the paper. Thirty-one papers (24.6%) had all taxa in the key diagnosed (Suppl. material 2, column AC) and this applied to 73 (37.4%) of the new genera and 364 of all genera (25.6%). An additional 18 (13.2%) papers had some of the previously described taxa diagnosed, primarily the genus considered most closely related to the new one such as when the new genus was being separated from the one to which it previously belonged. These were relevant to 37 (16.5%) of the new genera. While it might be expected that all genera were more likely to be diagnosed associated with keys to fewer genera, there was no significant difference in numbers of genera in keys with all, versus those with none, of the additional genera diagnosed (U = 1295.5, p > 0.9).

#### ﻿xii) Are habitus images provided?

Descriptions of nine new genera (6.1%) shared among four papers were not associated with habitus illustrations (Suppl. material 2, column AD) although images of at least parts of specimens of new genera were found in all but one. All the rest were associated with one or more high quality photographs except for four: one was indicated by SEM and three by line drawings.

### ﻿Overall key assessment

For all seventeen variables that could be assessed as binary, the average number of criteria that were suboptimal among the keys was 5.7 (SD = 1.99) with a range from 1 to 11 (Suppl. material 2 column AE). Because some features were not applicable to some keys, a more appropriate measure is the proportion of applicable criteria where a key was suboptimal, here the minimum was 7.7%, the maximum 68.8% and the average 36.1% (SD = 12.0%) (Suppl. material 2, column AF).

It is worth noting that these rather coarse evaluations are not likely to indicate the actual relative quality of the keys for a number of reasons including that all variables are treated as equally important, and the analysis does not incorporate the relative difficulty of identifying different taxa. However, the analysis does indicate the number of aspects that could have been made easier for the user.

## ﻿Discussion

Much has been said in recent years about crises in taxonomy and the impediment that lack of taxonomic information produces for other branches of science (most recently by Löbl et al. 2023). While there are suggestions to the contrary (e.g., Wheeler 2005: “Taxonomy does not exist to answer the question “What species is this?””) most taxonomists understand the importance of their work in making the world’s biota identifiable by others, at least at grant-writing time or in the introduction to manuscripts where they point out the importance of their research. Indeed, as Krell (2002) has stated “the most important impact of taxonomy is the usage of identification keys”. Given the considerable interest in insect identification for biodiversity survey work, conservation, biosecurity, and other applications, one might expect an ever-increasing need for relatively easy to use identification tools. Walter and Winterton (2007) noted the increased interest in insect identification, conducting a Google Search for “insect key” and found that the number of “hits” obtained increased by almost 20-fold in a single year (from 1,120 in March 2005 to 20,100 in March 2006). In April 2024, I found that the same search yielded “about 203,000,000” hits [though it should be noted that these search terms are highly imprecise]. While there is increasing accuracy of identifications based upon photographs (MacPhail et al. 2020) sometimes even for bulk samples (Fujisawa et al. 2023) and/or using convolutional neural network approaches (e.g., An et al. 2023), growing application of DNA barcode-based identifications (e.g., Gostel and Kress 2022), which also work for bulk samples (e.g., Gleason et al. 2023) not all people wishing to identify an insect want, or are able, to use these methods. Furthermore, newly described taxa will rarely be amenable to either of these approaches.

The number of new insect genera being described indicates that traditional aspects of taxonomic research remain essential and, of course, all identification methods associated with naming an organism require those names to be available in the first place (but see Ratnasingham and Hebert 2013). But making a name available is not the same as making it accessible. The standard way of making a newly available name accessible is through an identification key. Consequently, it was disappointing to find that a minority of the new genus descriptions was accompanied by a key that permitted identification and that when a key was provided, it rarely followed standard recommendations concerning effective key construction.

## ﻿Recommendations

Based upon the empirical assessment provided above, a set of best practices can be suggested for the description of new taxa dealing specifically with aspects of key construction. While my focus has been on keys associated with the description of new insect genera, these recommendations apply to any level in the taxonomic hierarchy for any taxonomic group. These recommendations are shown in Table 1, which also gives a partial rationale, cites an example for each best practice (when uncommon), and indicates whether adherence to the recommendation might be expected to take a large amount of research effort, journal space, or costs. How to adhere to these recommendations should be readily understood in most cases, although some additional explanation is given when this seems worth noting: such instances are denoted by an asterisk (*) in the first column of the table. The number at the beginning of a paragraph below the table refers to the recommendation number in the table.

**Table 1.** Recommendations concerning keys associated with new taxon descriptions, rationales for the recommendation, examples of best practices when these were uncommon and possible impediments to their implementation. Notes: * - see further discussion in recommendations section; Ψ - W much, w little- extra effort required; A much, a little - extra space required; $ much, ¢ little - extra cost likely involved; § - all keys in ZJLS.

Recommendation	Rationale	Example	Issues ψ
1. Include a key	At description, a new taxon will be best identified through use of a key. If it is an emendation to an earlier key either:		WA$
	a) put the couplet to the new genus near the very beginning or	Engel et al. 2022;	wa¢;
	b) state overtly and immediately above the key the features that should be present in a specimen for the key to be relevant.	Brunke 2021	wa¢
2.* Avoid placing a key in supplementary materials– unless	Supplementary materials often do not get as much attention from editors and reviewers as the rest of the text. IF they are necessary, put them in a stand-alone file with all necessary ease-of-use features as outlined below.		-A¢
3.* Make the key freely accessible	Open access identification tools will be seen, used and, perhaps most importantly, cited more often.		--$
4. Announce the presence of a key	Why hide the most useful part of your work from potential users? State the word “key” in the title, abstract, and/or keywords so it can be located using standard search methods.		---
5. Title the key appropriately	State the criteria for which the key is relevant: taxonomic group, geographic region, sex, caste: i.e., make it clear to a user whether the key will work for the specimen they wish to identify.		---
6. Diagnose the higher taxonomic group to which the key applies	State the features that make an individual belong to the taxonomic group dealt with in the key so users can tell easily whether they should use the key for a specimen at hand.		-a¢
7. Place that diagnosis usefully	Stating the features a specimen must possess for the key to be relevant immediately after the subheading to the key is most useful.	Prathapan and Konstantinov 2021	---
8.* Avoid monothetic couplets	Likely impossible to implement exhaustively, but at least try to avoid couplets that rely on a structure that is easily broken off (e.g., antennae) or requires dissection (e.g., genitalia).		Wa¢
9.* Make couplets decisive	Avoid couplets made entirely of features with exceptions or that are based on relative statements.		Wa$
10.* Avoid jargon	If a simpler synonymous term is available, use it.	Esteves and Fisher 2021	--- ;
	Illustrate what is meant, provide a glossary,	Wood et al. 2022	WA¢
	or cite a standard reference a user might be expected to have.		---
11.* Illustrate all leads	“A picture is worth a thousand words”. Many obscure features can be made perfectly clear through reference to an image.	Sharkey et al. 2021	WA$
12.* Place images within the key	Flipping backwards and forwards in a hardcopy or scrolling to and fro in a pdf can be extremely irritating. Hyperlinking is an excellent solution.	Sharkey et al. 2021; Colombo et al. 2022	WA$; w--
13. Allow retracing	Getting lost in a long key and having to go backwards or start from the beginning again wastes users’ time.	Xu et al. 2021	---
14*. Make keys as fan shaped as possible	Users are likely no less busy than are taxonomists and a fan-shaped key for 120 taxa will average an order of magnitude less effort to use than a comb-shaped one for the same taxa [but see points 17 and 18 below].	Bocakova et al. 2022	W--
15. Avoid leapfrogging	Leapfrog couplets are too easy to use incorrectly and are entirely unnecessary.		---
16. Avoid polychotomies… Unless	A user might not notice the third lead as almost all couplets are dichotomous, although:		---
	polychotomous couplets may be very efficient but IF used, format them to make their distinction from dichotomy obvious.	Sharkey et al. 2021	-a¢
17. Put easily identified or common taxa early in the key	If a few taxa in the key are abundant and the rest are relatively rare, placing them early in the key will save users’ time – I did not assess this formally as it was not possible to know which taxa were common/rare for all groups of insects assessed.	Packer et al. 2007	w--
18. Put taxa requiring complex manipulations late in the key	Faced with (for example) a key to 100 taxa in which the first couplet requires dissection of the genitalia, a newcomer to the group will likely decide to study something else. Not assessed: it was not always possible to know when complex manipulations were required (see 10 above).		w--
19. Diagnose all taxa that are keyed	A user can check their preliminary identification. It is best to include features that are in addition to those used in the identification key.	Gimmel and Leschen 2022	WA¢
20. Cite habitus images	As for 19, helps give users confidence and additional guidance. Almost all new genera were illustrated, so citing at least habitus figures in the key is easy and should be done.	Viswayjothi and Clark 2022	---
21.* Get naïve subjects to test the key	Those who write keys will understand what they mean, users might not. The best way to find out what parts are hard to follow is to get naïve subjects (i.e., not experts on the group) to test the key.		w--
22. Proofread the key particularly carefully	Some errors surely could not have been in the reviewed manuscript. Errors in couplet numbering and other formatting issues waste users’ time.		w--
23. Format the key so that it stands out clearly	Most keys had a heading in an easy-to-detect font, some were in text boxes, others were well hidden.	§ e.g., Hsiao and Pollock 2022	---

2. If a key is to be placed in supplementary materials, reviewers and editors should ensure that its quality is as good as it would have been if in the main body of the paper: many years of editorial experience convinces me that neither reviewers nor editors (myself included) routinely check supplementary materials as rigorously as they do the main body of the text. It is noteworthy that simple errors of couplet numbering and lack of contrast for specific features (Castro-Huertas et al. 2021) and a complete lack of figures (Xu et al. 2021) were found in both papers where keys were relegated to supplementary materials. It is perhaps telling that both were published in Systematic Entomology, the mostly highly ranked journal for insect taxonomic/systematics research. This illustrates the balance between highlighting long space-hungry keys and the exigencies of publishing where the number of available pages is limited.

3. Make the keys easily accessible. This is essential – papers behind a paywall will be inaccessible to too many potentially interested users. Maximally useful would be freely available, online depositories of emendable keys that authors can modify as new taxa are described. While clearly desirable, I am under no illusions that this is likely to become a commonly applied approach.

8. There is a balance between putting in a single feature that might not be assessable from a specimen requiring identification and putting in so many features that use of the key becomes unnecessarily time consuming. If possible, monothetic couplets should be moved towards the end of the key, or at least not used as the first couplet. If the number of features included in a couplet is large, they should be sequenced such that the earlier ones are easier to evaluate, and it should be stated that agreement with any of the features in the lead are sufficient for making a decision (or the combinations that have to be agreed should be stated).

9. No key should contain any couplets comprised only of qualified or relative statements. If there are exceptions to a particular feature, additional information should be provided that explains how taxa possessing the exception can be identified. For example, “compound eyes usually divergent below in frontal view, exceptions have the thorax entirely black” versus “compound eyes parallel-sided or convergent below and thorax pale-coloured.” Allied to this is the need not to leave alternatives implied – e.g., “distance between lateral ocelli wide (2× ocellar diameter)” versus “distance between lateral ocelli narrower” – state what the furthest apart the ocelli can be while still fitting the second option – is it 1.5× or 1.9×? If the latter, then the distinction might not be easy to make. Similarly, statements such as “carina between lateral ocelli present” are not useful unless the alternative is stated in the positive: “carina between lateral ocelli absent” might imply that this area is smooth, but a ridge between them or an area that is strongly sculptured might also be interpreted as being carinate. In both cases illustrations will help.

10. All branches of entomological taxonomy have their own terminologies, often inconsistent among different subgroups within individual higher-level taxa (e.g., use of the term scrobe by melittologists – it’s on the mesopleuron - versus its use by chalcidologists – it’s on the face) or even by different taxonomists studying exactly the same organisms. I made no attempt to assess how readily comprehensible the terminologies were as that would require familiarity with the words in standard usage in the description of most of the world’s biota. Nonetheless, it was abundantly clear that taxon-specific language was the norm and that despite my experience of using entomological keys for almost 50 years and teaching insect family level identification for more than 30, a frustratingly large proportion of keys contained couplets that I found incomprehensible without recourse to a glossary which was rarely provided or cited. However, some papers stood out for going beyond due diligence to make the work readily comprehensible. For example, Esteves and Fisher (2021) included detailed explanations of ant anatomy with all important features, including surface sculpture and setation, illustrated. Any interested novice should be able to use this key successfully. If not included in the paper, a glossary should be cited that explains the terminology being used, preferably one that is both readily available and with images to assist comprehension. Furthermore, authors should check that they are indeed using the terminology consistently with the cited reference. For example, it is common for entomologists to cite Harris (1979) when dealing with surface sculpture (1874 citations as of May 2024). Yet most melittologists use the term “striate” to refer to raised linear features whereas Harris (1979) clearly states that striae are depressed. Such deviations from defined usage should be stated explicitly.

11. Habitus images were ubiquitous, surely some features are shown in many of them such that they could be cited in the key. It is also useful to add a line pointing out where the key feature is, different colours can be used if multiple features are being shown in the same image. Indeed, it should be possible to produce language-neutral, image-driven identification keys (those in Marshall’s books on various insect orders, e.g., Marshall 2012, approach this, as do the keys of Sharkey et al. 2021, 2022).

12. Placing the images right next to the relevant couplet would frequently result in multiple copies of the same image arising in the paper. Thus, hyperlinking is likely the best option, especially when different images can come up on the screen simultaneously. Failing either of these options, placing all images that illustrate a key in one place (such as at the end of the paper, e.g., Yeshwanth and Konstantinov 2021) would be sensible as users can have two copies of the paper on the screen simultaneously and flip between the key in one and the images in the other.

14. Make keys as fan shaped as possible. This is relatively easy to do from a data matrix, such as one prepared for phylogenetic analysis or matrix-based key for which numerous automated procedures are available. Walter and Winterton (2007) listed 22 available early in 2006, noting that the list might not have been exhaustive even then. It seems that most of these are not in common use, nonetheless none of the papers surveyed used matrix keys.

In the absence of a matrix, it is possible to make a key more fan-like as follows (Packer et al. 2016). When there are couplets where one lead goes to one or a few taxa while the other goes to a much larger number, remove that couplet and see where the smaller number of taxa end up in the rest of the key and emend the downstream couplets accordingly. This process can be repeated as desired. Rigorous adherence to this recommendation is likely to take a lot of time for the researcher.

21. Authors will understand their key, reviewers are likely experts on the same or a similar group and will understand the key far better than will the average person that might want to use it. Colleagues or students who work with different taxonomic groups should be able to provide useful advice as to which parts of a key are problematic, where images are most essential and whether images that are provided point out the necessary morphological feature(s) clearly enough. This was Walter and Winterton’s (2007) recommendation #10. I did not assess this because it would not be possible to tell either whether anyone who was acknowledged for “commenting upon the manuscript” might have tested the key or whether they would be considered naïve users.

## ﻿Concluding remarks

Taxonomy is hard work and taxonomists are poorly funded (Britz et al. 2020), inadequately cited (Packer et al. 2018; Monckton et al. 2020), and negatively impacted by use of evaluation metrics that are not appropriate for the field (Krell 2000, 2002). Those that use the results of taxonomic research are also generally overworked and inadequately resourced. With the growth of activities such as citizen science (e.g., Gardiner and Roy 2022) and all-taxon biodiversity inventories (e.g., Ichter et al. 2022) an ever-increasing number of people are likely to want to identify a wide range of different insect taxa, but variance in their level of expertise will be considerable. While there are simple options for obtaining identifications, such as iNaturalist (e.g., Prudic et al. 2023), even those experts who perform research-quality determinations will generally have learned their skills by using keys. For new taxa, the paper describing them will be the only means of identification for some time. This suggests that whenever new taxa are described, the paper should include keys that are easily accessible, readily comprehensible, and easy to use. Yet my survey reveals that a minority of new genus descriptions were associated with a key to facilitate identification and, when provided, the keys rarely followed what should be standard protocols (Walter and Winterton 2007) to make them both usable and user friendly.

Some of the recommendations made above would be very time-consuming to implement (e.g., attempting to ensure a fan-shaped key structure) and others likely impossible (such as rigorous enforcement of non-monothetic couplets). Some are costly, for example page charges and/or journal production costs discourage duplication of images for ease of use in different parts of a manuscript. However, other issues associated with keys are extremely easy to deal with and are cost free: there is no reason to avoid making retracing in a key possible, there is no reason to have couplets that are misnumbered (or not numbered at all), there is no reason not to cite images in a key when they are already elsewhere in the manuscript.

Not all these problems are necessarily the responsibility of authors: journals will discourage repeating an image in a key when the same, or similar, image is also associated with a diagnosis or other aspects of the description of a new taxon. Sometimes a recommended component of a key is disallowed by a journal (e.g., editorial removal of information permitting retracing – Gibbs pers. comm. 1 May 2024; Onuferko, pers. comm. 4 July 2024). Sometimes the production team makes errors and/or authors are not given the opportunity to check the proofs (I have been the victim of this in a paper where one couplet was unreachable and one figure was empty white space because of production errors and the proofs being sent elsewhere).

Indeed, the potential for journals to improve the utility of taxonomy through outlining best practices for key construction in their instructions to authors seems entirely unrealized. For the six journals which published the largest number of papers describing new genera (Austral Entomology, EJT, SE, ZJLS, ZK, ZT) no guidelines on how to construct a key were provided. Three did not mention keys at all, one stated a condition where keys to species would be required while also noting that keys would be published with “high priority”, one stated where in the taxonomic section the key should be placed and a third stated that typesetting keys is difficult and showed how they should be written to make it easier for the production team. I have found only one journal that requires a key to be included when new taxa are described (the search among journals was not exhaustive). The same journal, the Canadian Entomologist, also had some information on how to construct a key (Canadian Entomologist 2024). Ironically, this journal published no new genera during the time period I considered.

It is illustrative to compare the complete absence of guidelines on key construction to the enormous amount of space used instructing authors how to format references, a situation where all formats are likely equally comprehensible to any user. Guidance on how to construct a key is clearly a more pressing problem given that all keys fell foul of at least some of the issues noted above. Journals should add guidelines for key construction to their instructions to authors. Requirements that descriptions of new taxa should be accompanied by a key should be standard editorial practice, keys should be reviewed as carefully as possible to ensure they follow best practices and, unless hyperlinking to figures occurs, publishers should permit the formatting that is necessary to make keys easy to use even if this means that some figures are duplicated and/or some pages have extra empty space.

## ﻿Additional information

### Author ORCIDs

Laurence Packer https://orcid.org/0000-0002-1711-8203

### Data availability

All of the data that support the findings of this study are available in the main text or Supplementary Information.

## Supplementary materials

Supplementary material 1 Complete list of papers that included keys used in the analyses Data type docx https://binary.pensoft.net/file/1152309This dataset is made available under the Open Database License (http://opendatacommons.org/licenses/odbl/1.0/). The Open Database License (ODbL) is a license agreement intended to allow users to freely share, modify, and use this Dataset while maintaining this same freedom for others, provided that the original source and author(s) are credited.Laurence Packer

Supplementary material 2 Data extracted from assessed identification keys Data type xlsx https://binary.pensoft.net/file/1152310This dataset is made available under the Open Database License (http://opendatacommons.org/licenses/odbl/1.0/). The Open Database License (ODbL) is a license agreement intended to allow users to freely share, modify, and use this Dataset while maintaining this same freedom for others, provided that the original source and author(s) are credited.Laurence Packer
