# Supplementary material for: ﻿A survey of keys for the identification of newly described insect genera: recommendations for authors, reviewers, editors, and publishers
Source: Zookeys. 2024 Oct 11;1215:65–90. doi: 10.3897/zookeys.1215.130416 (PMC11489712; doi:10.3897/zookeys.1215.130416)
Supplement: Supplementary material 1 — Complete list of papers that included keys used in the analyses [file zookeys-1215-065_article-130416__-s001.docx]

| References |
| --- |
| 1. Allsopp PG (2022) Australian Melolonthini (Coleoptera: Scarabaeidae: Melolonthinae): reclassification of eight species to *Antitrogus* Burmeister, 1855 and *Barryfilius* new genus. Zootaxa 5213: 513–545. |
| 2. Amina P, Rajmohana K, Dinesh KP, Asha G (2022) Integrative taxonomic studies on *Rinacapritermes* Amina & Rajmohana, n. gen. (Blattodea: Isoptera: Termitidae) with two new species from India. Zoosystema 44: 109–124. |
| 3. Anderson RS, Bermudez Higinio MF ((2022) *Koreguajus antennatus* Anderson and Bermúdez Higinio, a new genus and new species of Sphenophorina (Coleoptera: Curculionidae: Dryophthorinae; Rhynchophorini) from Colombia. Zootaxa 5190: 595–600. |
| 4. Ardila-Camacho A, Rivera-Gasperín SL, Martins CC, Contreras-Ramos A (2021) A reappraisal of the taxonomy of Neotropical Sialidae (Insecta: Megaloptera): with the description of a new genus from Cuba. European Journal of Taxonomy 782: 21–54. |
| 5. Balkenohl M (2022) Revision of the genus *Parasyleter* Balkenohl, 2021 with the descriptions of four new species (Coleoptera: Carabidae: Clivinini). Revue suisse de Zoologie 129: 229–241. |
| 6. Balkenohl M (2022) On the female gonocoxites of Asian, Arabian, and African Clivinini with realignment of the *Thliboclivina*-, *Physoclivina*-, and *Eoclivina*-species groups, and notes on the higher systematics of the tribe Clivinini Rafinesque, 1815 (Coleoptera, Carabidae). Zootaxa 5190: 99–126. |
| 7. Ballantyne L, Kawashima I, Jusoh WF, Suzuki H (2022) A new genus for two species of Japanese fireflies having aquatic larvae (Coleoptera, Lampyridae) and a definition of *Luciola* s. str. European Journal of Taxonomy 855: 1–54. |
| 8. Barr CB, Shepard WD (2021) A review of the Larainae of Australia with description of seven new species and the new genus *Australara* (Coleoptera, Byrrhoidea, Elmidae). ZooKeys 1073: 55–117. |
| 9. Bartlett JS, Lambkin CL (2022) Australian Opilonini (Coleoptera: Cleridae: Clerinae) part I: A revised taxonomy for Australian *Opilo* Latreille including descriptions of new genera and species. Zootaxa 5220: 1–81. |
| 10. Basílio DS, Vaz-De-Mello FZ, Almeida LM (2022) A new genus and species of Neotropical Hybosorinae Erichson, 1847 (Coleoptera: Hybosoridae). Anais da Academia Brasileira de Ciências 94 suppl 3 e20201846. |
| 11. Belokobylskij SA, Zaldívar-Riverón A (2021) Reclassification of the doryctine tribe Rhaconotini (Hymenoptera, Braconidae). European Journal of Taxonomy 741: 1–168. |
| 12. Benda D, Pohl H, Nakase Y, Beutel R, Straka J (2022) A generic classification of Xenidae (Strepsiptera) based on the morphology of the female cephalothorax and male cephalotheca with a preliminary checklist of species. ZooKeys 1093: 1–134. |
| 13. Bezark LG, Santos-Silva A (2022) Description of a new genus and three new species of Rhopalophorini (Coleoptera: Cerambycidae: Cerambycinae) from Ecuador. The Pan-Pacific Entomologist 97: 226–239. |
| 14. Bilton DT, Shepard WD (2022) *Rhithrops capensis* gen. et sp. nov., a new aquatic dryopid beetle from the Western Cape of South Africa (Coleoptera: Dryopoidea: Dryopidae). Zootaxa 5195: 539–553. |
| 15. Bocakova M, Campello-Gonçalves L, Da Silveira LFL (2022) Phylogeny of the new subfamily Cladodinae: neotenic fireflies from the Neotropics (Coleoptera: Lampyridae). Zoological Journal of the Linnean Society 195: 1181–1199. |
| 16. Boudinot BE, Borowiec ML, Prebus MM (2022) Phylogeny, evolution, and classification of the ant genus *Lasius*, the tribe Lasiini and the subfamily Formicinae (Hymenoptera: Formicidae). Systematic Entomology 47: 113–151. |
| 17. Brunke AJ (2021) New relictual genera in Cyrtoquediini and Indoquediini (Coleoptera: Staphylinidae: Staphylininae). ZooKeys 1076: 109–124. |
| 18. Camacho GP, Franco W, Branstetter MG, Pie MR, Longino JT, Schultz TR, Feitosa RM (2022) UCE phylogenomics resolves major relationships among ectaheteromorph ants (Hymenoptera: Formicidae: Ectatomminae, Heteroponerinae): a new classification for the subfamilies and the description of a new genus. Insect Systematics and Diversity 6: 1–20 |
| 19. Camargo A, Vieira R, Fisher E (2022). A unique robber fly species and genus of Asilinae from Argentina (Diptera: Asilidae). Austral Entomology 61: 407–419. |
| 20. Castro‐Huertas V, Forero D, Grazia J (2021) Evolution of wing polymorphism and genital asymmetry in the thread‐legged bugs of the tribe Metapterini Stål (Hemiptera, Reduviidae, Emesinae) based on morphological characters. Systematic Entomology 46: 28–43. |
| 21. Chamorro M L, Presnall TM (2022) *Randersonia* Chamorro, a new genus with two new species of North American Cryptorhynchinae (Coleoptera: Curculionidae). Zootaxa 5222: 591–599. |
| 22. Chen J, Li Z, Jiang L, Qiao G (2021) *Glyphochaitophorus*, a new genus of Chaitophorini (Hemiptera: Aphididae: Chaitophorinae) from China. Zootaxa 4975: 581–591. |
| 23. Chen X, Freidberg A, Islam MS, Zhu C (2021) Phylogeny of Adramini (Diptera, Tephritidae) based on integrative evidence. Zoologica Scripta 50: 71–83. |
| 24. Colombo WD, Tribull CM, Waichert C, Azevedo CO (2022) Integrative taxonomy solves taxonomic impasses: a case study from Epyrinae (Hymenoptera, Bethylidae). Systematic Entomology 47: 504–529. |
| 25. Costa LO, Duarte PR, Iannuzzi L, Grossi PC (2022) Taxonomic revision and notes on natural history of the enigmatic beetle genus *Gibboryctes* Endrödi (Coleoptera: Melolonthidae: Dynastinae). Journal of Natural History 56: 191–225. |
| 26. Cumming RT, Bank S, Bresseel J, Constant J, Le Tirant S, Dong Z, Sonet G, Bradler S (2021) *Cryptophyllium*, the hidden leaf insects–descriptions of a new leaf insect genus and thirteen species from the former celebicum species group (Phasmatodea, Phylliidae). ZooKeys 1018: 1–179. |
| 27. Cumming RT, Le Tirant S (2022) Three new genera and one new species of leaf insect from Melanesia (Phasmatodea, Phylliidae). ZooKeys 1110: 151–200. |
| 28. Ćurčić S, Pavićević D, Vesović N, Vrbica M, Kuraica M, Marković Đ, Petković M, Lazović V, Pantelić D, Bosco F (2021) On the diversity of subterranean beetles of the Dinarides: new leiodid taxa (Coleoptera: Leiodidae) from Serbia. European Journal of Taxonomy 782: 55–81. |
| 29. David KJ, Hancock D, Gracy RG, Sachin K (2022) A new genus of fruit fly in subfamily Dacinae (Diptera: Tephritidae) from India. Zootaxa 5195: 585–597. |
| 30. David KJ, Hancock DL, Han HY, Gracy GR, Sachin K, Swathi RS (2021) A new genus in the tribe Acidoxanthini (Diptera: Tephritidae: Trypetinae) from India, with a discussion of its phylogenetic relationships. Journal of Asia-Pacific Entomology 24: 1194–1201. |
| 31. Davidian GE (2022) A new genus and new species of the weevils (Coleoptera, Curculionidae: Entiminae) from the Sino-Tibetan Mountains with a key to Asian genera of Blosyrini. Entomological Review 102: 697–717. |
| 32. Davidian GE (2021) A new weevil genus closely related to *Dactylotinus* Korotyaev, 1996 (Coleoptera, Curculionidae: Entiminae) with new species from the Sino-Tibetan Mountains. Entomological Review 101: 992–1003. |
| 33. Dellapé G, Fuentes D (2021) *Jocezia*, a new Carpocorini genus from the Neotropics (Heteroptera: Pentatomidae: Pentatominae). Zootaxa 4958: 503–509. |
| 34. Dietrich CH (2021) New species of *Mayawa* Fletcher and description of a related new Australian leafhopper genus (Hemiptera: Cicadellidae: Deltocephalinae: Paralimnini). Zootaxa 4933: 575–585. |
| 35. Engel MS, Nguyen LTP, Tran NT, Truong TA, Motta AFH (2022) ﻿A new genus of minute stingless bees from Southeast Asia (Hymenoptera, Apidae). ZooKeys 1089: 53–72. |
| 36. Esteves FA, Fisher BL (2021) *Corrieopone nouragues* gen. nov., sp. nov., a new Ponerinae from French Guiana (Hymenoptera, Formicidae). ZooKeys 1074: 83–173. |
| 37. Flórez-V C (2022) A new genus and a new species of treehopper (Hemiptera: Membracidae) from the páramo of Tatamá in Colombia. Zootaxa 5195: 143–154. |
| 38. Gaimari SD, Havill NP (2021) A new genus of Chamaemyiidae (Diptera: Lauxanioidea) predaceous on Adelgidae (Hemiptera), with a key to chamaemyiid species associated with Pinaceae-feeding Sternorrhyncha. Zootaxa 5067: 1–39. |
| 39. García K, Botero JP, Santos-Silva A (2021) A new genus of Achrysonini and new records in Cerambycidae (Coleoptera, Chrysomeloidea) from Colombia. European Journal of Taxonomy 735: 89–109. |
| 40. Giachino PM, Eberhard, Serina G (2021) A rich fauna of subterranean short-range endemic Anillini (Coleoptera, Carabidae, Trechinae) from semi-arid regions of Western Australia. ZooKeys 1044: 269–337. |
| 41. Gilasian E, Ziegler J, Tóthová A, Parchami-Araghi M (2021) A new genus and species of tachinid flies from Iran (Diptera, Tachinidae, Goniini). European Journal of Taxonomy 746: 162–185. |
| 42. Gimmel ML, Leschen RA (2022) Revision of the genera of Picrotini (Coleoptera: Cryptophagidae: Cryptophaginae). Acta Entomologica Musei Nationalis Pragae 62: 61–109. |
| 43. Girón JC, Short AEZ (2021) The Acidocerinae (Coleoptera, Hydrophilidae): taxonomy, classification, and catalog of species. ZooKeys 1045: 1–236. |
| 44. Gonçalves RB (2021) A revised genus-level classification for the Neotropical groups of the cleptoparasitic bee tribe Sphecodini Schenck (Hymenoptera, Apidae, Halictinae). Revista Brasileira de Entomologia 65: 1–10. |
| 45. Haran J, Benoit L, Procheş Ş, Kergoat GJ (2022) *Ebenacobius* Haran, a new southern African genus of flower weevils (Coleoptera: Curculioninae: Derelomini) associated with dicotyledonous plants. European Journal of Taxonomy 818: 1–54. |
| 46. He Z, Zhang T, Ma L (2021) Crickets of subfamily Nemobiinae Saussure, 1877 (Orthoptera: Grylloidea; Trigonidiidae) from China with descriptions of new genera and new species. Zootaxa 5011: 1–70. |
| 47. Herrera-Florez AF (2021) *Dorothee*, a new genus of Cryptinae (Hymenoptera: Ichneumonidae) from Peru. Zootaxa 5005: 587–595. |
| 48. Hlaváč P (2021) A revision of the New Zealand Tyrini (Coleoptera, Staphylinidae, Pselaphinae), with the description of two new genera and five new species. New Zealand Entomologist 44: 38–58. |
| 49. Hossain MS, Kwon JH, Suh S J, Kwon Y J (2021) A remarkable microleafhopper genus *Koreoneura* gen. nov. and newly recorded genus of the tribe Dikraneurini from Korea (Homoptera: Cicadellidae: Typhlocybinae). Zootaxa 4951: 353–360. |
| 50. Hsiao Y, Pollock DA (2022) Morphology-based phylogeny of oval palm and flower beetles (Coleoptera: Mycteridae: Eurypinae), with descriptions of new genera and species from Australia. Zoological Journal of the Linnean Society 196: 677–703. |
| 51. Huang XD, Chen XS, Yang L, Long JK (2022) *Gigasanalis*, a new genus of the tribe Achilini with the description of a new species from China (Hemiptera, Fulgoromorpha, Achilidae). European Journal of Taxonomy 852: 85–97. |
| 52. Hugel S, Warren BH, Desutter-Grandcolas L (2021) The Phalangopsidae crickets (Orthoptera, Grylloidea) of the Seychelles Archipelago: Taxonomy of an ecological radiation. Zootaxa 5047: 201–246. |
| 53. Huo QB, Du Y-Z (2021) A new genus of Isoperlinae (Plecoptera: Perlodidae) from Tibet.  Zootaxa 4996: 343–352. |
| 54. Huo KK, Zhao L, Mengual X, Li G, Liu X, Zhao LJ, Chen ZN (2022) *Biema* Huo & Zhao gen. nov., a new flower fly genus (Diptera, Syrphidae) from China. European Journal of Taxonomy 852: 98–116. |
| 55. Jałoszyński P, Ślipiński A (2022) Revision of the family Murmidiidae (Coleoptera: Coccinelloidea). Zootaxa 5109: 1–102. |
| 56. Jałoszyński P (2021) *Sinonichnus* gen. n. for *Scydmoraphes yunnanensis* Jałoszyński, and description of *Sinonichnus leiodicornutus* sp. n.(Coleoptera, Staphylinidae, Scydmaeninae). Zootaxa 4938: 487–496. |
| 57. Jennings JT, Macdonald JA, Schiff NM, Parslow BA (2021) Revision of Xiphydriinae (Hymenoptera: Xiphydriidae) of Australia. Austral Entomology 60: 631–658. |
| 58. Jordal BH (2021) Molecular and morphological revision of Afrotropical Hypoborini (Coleoptera: Curculionidae: Scolytinae) revealed novel bark beetle taxa with narrow geographical distributions. European Journal of Entomology 118: 90–110. |
| 59. Jordal BH (2021) A phylogenetic and taxonomic assessment of Afrotropical Micracidini (Coleoptera, Scolytinae) reveals a strong diversifying role for Madagascar. Organisms Diversity and Evolution 21: 245–278. |
|  |
| 61. Kirejtshuk AG, Kovalev A V (2022) Monograph on the Cillaeinae (Coleoptera: Nitidulidae) from the Australian Region with comments on the taxonomy of the subfamily. Zootaxa 5103: 1–133. |
| 62. Kluge N J, Vasanth M, Balasubramanian C, Sivaramakrishnan KG (2022) Review of the Kimminsula–complex (Ephemeroptera, Leptophlebiidae). Zootaxa 5212: 1–140. |
| 63. Kolencik S, Sychra O, Allen JM (2021) Another puzzle piece in the systematics of the chewing louse genus *Myrsidea*, with a description of a new genus *Apomyrsidea*. European Journal of Taxonomy 748: 36–50. |
| 64. Krivokhatsky V, Dobosz R, Ábrahám L (2022) The new antlion genus *Dominikon* gen. nov. with the description of a new species, *Dominikon aspoecki* sp. nov. (Neuroptera: Myrmeleontidae). Zootaxa 5196: 94–114. |
| 65. Lee GE, Han T, Park H, Qi M, Li H (2021) A phylogeny of the subfamily Thiotrichinae (Lepidoptera: Gelechiidae) with a revision of the generic classification based on molecular and morphological analyses. Systematic Entomology 46: 357–379. |
| 66. Lee YJ (2022) A new genus and species of the subtribe Leptopsaltriina (Hemiptera: Cicadidae: Leptopsaltriini) from Sabah, Malaysia. Journal of Asia-Pacific Biodiversity 15: 459–462. |
| 67. Legalov AA (2021) A new genus of the tribe Rhinocartini (Coleoptera, Rhynchitidae) from the Solomon Islands. Ecologica Montenegrina 49: 54–58. |
| 68. Li T, Sun SP, Sheng ML (2022) A new genus and species of Ctenopelmatinae (Hymenoptera, Ichneumonidae) from China. Journal of Hymenoptera Research 92: 199–210. |
| 69. Liu LY, Sittichaya W (2022) The Oriental genera of Xyloperthini (Coleoptera: Bostrichidae: Bostrichinae), with a new genus and species from Thailand, and a key to the genera. European Journal of Taxonomy 828: 45–60. |
| 70. Liu YJ, Xu JY, He ZQ (2021) Report of a new genus *Majialandrevus*, with a new species *M. dingguo* from Western Yunnan, China (Orthoptera: Gryllidae: Landrevinae). Zootaxa 4985: 137–141. |
| 71. López-Pérez S, Zaragoza-Caballero S (2021) Two new genera of Telegeusidae (Coleoptera) from Mexico. Revista Mexicana de Biodiversidad 92: 1–10. |
| 72. Lucañas CC (2021) *Bundoksia* gen. nov.(Dictyoptera: Blattodea: Blattidae), a new sexually dimorphic cockroach from the Philippines. Journal of Natural History 55: 1009–1020. |
| 73. Luo JY, Gong QB, Xie Q (2022) ﻿ A new genus and species of minute litter bugs family Schizopteridae Reuter, 1891 from China (Hemiptera, Heteroptera, Dipsocoromorpha). ZooKeys 1120: 177–193. |
| 74. Macgowan I (2022) A new genus within the tribe Lonchaeini (Diptera, Lonchaeidae) based on two species from the Neotropics. Zootaxa 5182: 559–566. |
| 75. Machado RJP, Martins CC, Aspöck H, De Miranda Tavares LG, Aspöck U (2022) The first cave associated genus of Berothidae (Insecta: Neuroptera), and a new interpretation of the subfamily Cyrenoberothinae. Zoological Journal of the Linnean Society 195: 1422–1444. |
| 76. Macià R, Ylla J, Gastón J, Huertas M, Bau J (2022) The species of *Eilema* Hübner,[1819] *sensu* *lato* present in Europe and North Africa (Lepidoptera: Erebidae: Arctiinae: Lithosiini). Zootaxa 5191: 1–87. |
| 77. Meregalli M, Borovec R, Cervella P, Santovito A, Toševski I, Ottati S, Nakládal O (2021) The Namaini, a new weevil tribe with six new genera from South Africa (Coleoptera: Curculionidae: Entiminae). Zoological Journal of the Linnean Society 193: 95–123. |
| 78. Miller DR, Stocks IC (2022) New genera and species of felt scales (Hemiptera: Coccomorpha: Eriococcidae), with descriptions of new species and immature instars of described species. Zootaxa 5221: 1–213. |
| 79. Moir ML (2022) Revision of the lacebug tribe Ceratocaderini (Hemiptera: Tingidae). Austral Entomology 61: 277–301. |
| 80. Mondragón-F SP, Morales I, Moreira FF (2021) *Telmatometropsis fredyi* gen. nov., sp. nov.: a new water strider from the Colombian Pacific region (Insecta, Hemiptera, Gerridae). ZooKeys 1043: 87–102. |
| 81. Namaki-Khameneh R, Khaghaninia S, Disney RHL, Maleki–Ravasan N (2021) The scuttle flies (Diptera: Phoridae) of Iran with the description of *Mahabadphora aesthesphora* as a new genus and species. PLOS one, 16 p.e0257899. |
| 82. Namaki-Khameneh R, Khaghaninia S, Disney RHL, Maleki-Ravasan N (2021) Nine new species of scuttle flies, including one new genus (Diptera: Phoridae) from Iran. Biologia 76: 2895–2912. |
| 83. Nascimento FEDL, Santos-Silva A (2021) The interesting and complex tribe Oxycoleini (Coleoptera, Cerambycidae): a new genus, new species, and taxonomic notes. Zootaxa 5060: 353–370. |
| 84. Nieves-Aldrey JL (2022) Description of *Fumariphilus* Nieves-Aldrey, gen. nov., a new genus of herb gall wasps, with a key to genera of the tribe Aulacideini (Hymenoptera: Cynipidae). Zootaxa 5155: 393–413. |
| 85. Pacheco TL, Monné ML, Vaz de Mello FZ, Ahrens D (2022) First non-feeding Sericini beetles (Coleoptera, Scarabaeidae): new genus from Amazonia and phylogenetic position. Organisms Diversity and Evolution https://doi.org/10.1007/s13127-022-00555-x. |
| 86. Pal S, Kumar V, Panjaliya RK, Tyagi K (2022) A new genus and species of subfamily Dendrothripinae (Thysanoptera: Thripidae) from India. Zootaxa 5175: 383–388. |
| 87. Perreau M (2021) The near Eastern species of Leptodirini (Coleoptera: Leiodidae: Cholevinae). Annales de la Société entomologique de France (NS) 57: 289–312. |
| 88. Pham T H, Lee YJ (2021) A new genus and species of the subtribe Aolina (Hemiptera: Cicadidae: Dundubiini) from Vietnam, with discussion on the taxonomic positions of *Meimuna*-like genera and species groups. Journal of Asia-Pacific Entomology 24: 175–181. |
| 89. Pham TH, Lee YJ (2021) A new genus and species of the subtribe Leptopsaltriina (Hemiptera: Cicadidae: Leptopsaltriini) from Vietnam, with a key to the genera of Leptopsaltriina. Journal of Asia-Pacific Entomology 24: 1202–1205. |
| 90. Polak S, Mulaomerović J (2021) *Rudogorites simonei* gen. nov. and sp. nov. from Central Bosnia (Coleoptera: Leiodidae: Cholevinae: Leptodirini). Zootaxa 5061: 545–558. |
| 91. Polhemus D (2021) *Callivelia*, a new genus for certain Neotropical Veliinae (Heteroptera: Veliidae), including description of a new species. Zootaxa 4950: 345–360. |
| 92. Polhemus DA (2022) Two new genera and six new species of terrestrial Hydrometridae (Hemiptera: Heteroptera) from French Polynesia. Zootaxa 5190: 69–98. |
| 93. Prathapan KD, Konstantinov AS (2021) Revision of Oriental flea beetle genera with subparallel intercoxal ridges on the first abdominal ventrite (Coleoptera, Chrysomelidae, Galerucinae, Alticini). Journal of Natural History 55: 1521–1598. |
| 94. Ranjith AP, Priyadarsanan DR (2022) A new Darwin wasp genus, *Soliga* (Hymenoptera: Ichneumonidae: Metopiinae), from India. European Journal of Taxonomy 852: 57–76. |
| 95. Ranjith AP, Priyadarsanan DR (2022) *Atree*, a remarkable new genus of the subfamily Brachistinae (Hymenoptera: Braconidae) and the first report of the tribe Diospilini from India. Zootaxa 5105: 571–580. |
| 96. Ranjith AP, Samartsev KG, Nasser M (2021) Discovery of the chelonine tribe Adeliini Viereck, 1918 (Hymenoptera: Braconidae) from the Indian subcontinent with the description of a new genus from south India. Zootaxa 4926: 1–25. |
| 97. Ruta R (2021) Three new genera of large marsh beetles (Coleoptera: Scirtidae) from Valdivian temperate rain forests of southern South America. Zootaxa 5048: 451–485. |
| 98. Ruta R, Yoshitomi H (2022) *Calvariomorphus*—a new genus of marsh beetles (Coleoptera: Scirtidae) with remarkable elytral excitators. Zootaxa 5120: 65–82. |
| 99. Saigusa T, Sinclair BJ (2022) A new genus of Clinocerinae (Diptera: Empididae) from Taiwan and Yunnan (China). Bonn Zoological Bulletin 71:177–183. |
| 100. Sanborn AF (2021) The cicadas (Hemiptera: Cicadoidea: Cicadidae) of Madagascar including a new tribe, five new genera, twelve new species, four new species synonymies, five revised species status, ten new combinations, new tribal assignments for four genera, one new subtribe synonymy, a checklist and key to the species. Zootaxa 4937: 1–79. |
| 101. Santis MD (2022) *Dolichozelia* gen. nov., a new genus of Dexiini (Diptera: Tachinidae) from Brazil. Austral Entomology 61: 302–311. |
| 102. Santos-Silva A, García K, Botero JP (2021) A review of the history of the names *Hamaticherus* Dejean and Plocaederus Dejean and description of a new genus and species (Coleoptera: Cerambycidae: Cerambycinae). Insecta Mundi 887: 1–32. |
| 103. Schawaller W, Bigalk S (2021) Two new genera and six new species of Stenosini (Coleoptera: Tenebrionidae: Pimeliinae) from Myanmar (Burma), collected by René Fouquè. Zootaxa 5068: 133–141. |
| 104. Setliff G, Pancini L, Bramanti A (2021) Review of *Eudyasmus*, with descriptions of a new species from Waigeo Island, Indonesia, and a closely related new genus (Coleoptera: Curculionidae, Molytinae, Eudyasmini). Fragmenta Entomologica 53: 377–390. |
| 105. Sharkey MJ, Baker A, Manjunath R, Hebert PD (2022) ﻿Description of *Chilearinus* Sharkey gen. nov. and status of Nearctic *Earinus* Wesmael, 1837 (Braconidae, Agathidinae) with the description of new species. ZooKeys 1099: 57–86. |
| 106. Sharkey MJ, Janzen DH, Hallwachs W, Chapman EG, Smith MA, Dapkey T, Brown A, Ratnasingham S, Naik S, Manjunath R, Perez K (2021) Minimalist revision and description of 403 new species in 11 subfamilies of Costa Rican braconid parasitoid wasps, including host records for 219 species. ZooKeys 1013: 1–665. |
| 107. Shimizu A, Pitts JP, Rodriguez J, Wahis R, Yoshimura J (2021) Systematics and convergent evolution in three Australian genera of Pepsinae spider wasps (Hymenoptera: Pompilidae). Austral Entomology 60: 301–316. |
| 108. Sinclair BJ (2021) A new winter dance fly genus from California (Diptera: Empidoidea: Brachystomatidae). Proceedings of the Entomological Society of Washington 123: 622–637. |
| 109. Sinclair BJ, Saigusa T (2022) A new aquatic associated genus of Trichopezinae from the Southern Hemisphere (Diptera: Empidoidea: Brachystomatidae). Records of the Australian Museum 74: 75–98. |
| 110. Sinclair BJ, Shamshev IV, (2021) World revision of *Iteaphila* with unbranched radial vein (Diptera: Empidoidea: Iteaphilidae). Zootaxa 4968: 1–89. |
| 111. Singh LRK, Achterberg CV, Sheela S (2022) Studies on the subfamily Exothecinae (Hymenoptera: Braconidae) with the description of a new genus and a new species from India. Zootaxa 5133: 40–52. |
| 112. Ślipiński A, Lawrence JF, Escalona HE (2021) The genera of Inopeplinae (Coleoptera: Salpingidae), world generic key, descriptions of four new genera and revision of the Australian fauna. Annales Zoologici 71: 701–735. |
| 113. Souma J, Kamitani S (2021) Taxonomic review of the lace bug genus *Omoplax* (Hemiptera: Heteroptera: Tingidae) endemic to “Oriental Galapagos” (the Ogasawara Islands, Japan) with the description of its new allied genus and species. Entomological Science 24: 3–11. |
| 114. Storozhenko SY (2021) On the exact position of the tribe Incolacridini in the modern classification of grasshoppers (Orthoptera: Acrididae). Zootaxa 4970: 106–118. |
| 115. Sublett CA, Cook JL (2021) A comprehensive revision of the genus Metaparia Crotch, 1873 (Coleoptera: Chrysomelidae: Eumolpinae) and description of a new genus. The Coleopterists Bulletin 75: 779–811. |
| 116. Tan MK, Malem J, Legendre F, Dong J, Baroga‐Barbecho JB, Yap SA, Wahab RBHA, Japir R, Chung AY, Robillard T (2021) Phylogeny, systematics and evolution of calling songs of the Lebinthini crickets (Orthoptera, Grylloidea, Eneopterinae), with description of two new genera. Systematic Entomology 46: 1060–1087. |
| 117. Tauber CA (2021) The New World Belonopterygini (Neuroptera: Chrysopidae): descriptions of a new genus and species from the West Indies and comparisons among the genera. Zootaxa 4975: 509–543. |
| 118. Trzna M, Baa P (2021) Description of a new genus and species, *Portentus marschneri* (Coleoptera: Anthribidae), from Penang National Park, Malaysia. Zootaxa 5004: 551–563. |
| 119. Tshernyshev SE (2021) *Pectotibialis paghmanensis* Tshernyshev gen. nov.–a new genus and species of soft-winged flower beetle (Coleoptera, Cleroidea, Malachiidae) from Afghanistan. European Journal of Taxonomy 775: 1–14. |
| 120. Tshernyshev SE (2021) The revision of soft-winged flower beetle genus *Dicranolaius* Champion, 1921 (Coleoptera: Cleroidea: Malachiidae) with description of a new genus Australolaius gen. n. from Australia. Invertebrate Zoology 18: 159–176. |
| 121. Tshernyshev SE (2022) Soft-winged flower beetles (Coleoptera: Malachiidae) of the United Arab Emirates. European Journal of Taxonomy 817: 58–77. |
| 122. Vega-Badillo V, Cancino-López RJ, Martins CC, Contreras-Ramos A (2022) A remarkable new genus and species of wedge-shaped beetle from Volcán Tacaná, Mexico (Coleoptera: Ripiphoridae: Ripidiinae). Biologia 77: 479–488. |
| 123. Viswajyothi K, Clark S M (2022) New World genera of Galerucinae Latreille, 1802 (tribes Galerucini Latreille, 1802, Metacyclini Chapuis, 1875, and Luperini Gistel, 1848): an annotated list and identification key (Coleoptera: Chrysomelidae). European Journal of Taxonomy 842: 1–102. |
| 124, Wanat M (2021) New basal taxa of South African Apioninae (Coleoptera: Curculionoidea: Brentidae). Zootaxa 5035: 1–60. |
| 125. Wood TJ, Patiny S, Bossert S (2022) ﻿An unexpected new genus of panurgine bees (Hymenoptera, Andrenidae) from Europe discovered after phylogenomic analysis. Journal of Hymenoptera Research 89: 183–210. |
| 126. Worthy R, González JM, Zilli A (2022) A review of the genera *Amauta* Houlbert, 1918 and *Divana* JY Miller, 1982 (Lepidoptera: Castniidae) with description of a new genus. Zootaxa 5194: 301–342. |
| 127. Xu Y, Dietrich CH, Zhang YL, Dmitriev DA, Zhang L, Wang YM, Lu SH, Qin DZ (2021) Phylogeny of the tribe Empoascini (Hemiptera: Cicadellidae: Typhlocybinae) based on morphological characteristics, with reclassification of the Empoasca generic group. Systematic Entomology 46: 266–286. |
| 128. Yan, B., He, H.L., Yang, M.F. and Webb, M.D., (2021) A new genus and species of Mileewini leafhoppers (Hemiptera, Cicadellidae, Mileewinae) from China, with a key to genera. ZooKeys 1028: 61–67. |
| 129. Yan YC, Yan WL, Deng TJ, Wei MC (2022) ﻿ *Asicimbex* Yan, Deng & Wei, a new genus with eight new species and four new combinations (Hymenoptera, Cimbicidae). Journal of Hymenoptera Research 91: 265–308. |
| 130. Yasunaga T, Chérot F, Schwartz MD (2021) New genera and species of the Oriental mirine plant bugs from Southeast Asia, with six new combinations (Insecta: Heteroptera: Miridae: Mirinae: Mirini). Raffles Bulletin of Zoology 69: 137–155. |
| 131. Yeshwanth HM, Konstantinov FV (2021) Review of the plant bug tribe Eccritotarsini (Hemiptera: Heteroptera: Miridae) of India and Sri Lanka with description of two new genera and six new species. European Journal of Taxonomy 745: 1–69. |
| 132. Yin,Z, Kurbatov SA (2022) *Schmidtinus annapurnensis*, a new genus and species of Batrisitae from Nepal (Coleoptera: Staphylinidae: Pselaphinae). Zootaxa 5169: 79–86. |
| 133. Zahniser JN (2021) Revision of the New World leafhopper tribe Faltalini (Hemiptera: Cicadellidae: Deltocephalinae) and the evolution of brachyptery. Zootaxa 4954: 1–160. |
| 134. Zahradník P, Háva J (2022) *Tanzaptinus ornatus* gen. n.(coleoptera: ptinidae: ptininae), a new genus and species of spider beetles from Tanzania. Euroasian Entomological Journal 21: 75–77. |
| 135. Zettel H, Laciny A (2021) The taxonomy of some unusual Microveliinae (Hemiptera: Heteroptera: Veliidae) from India. Acta Entomologica Musei Nationalis Pragae 61: 517–527. |
| 136. Zhang Y-F, Meng L-Z, Beaver RA (2022) A review of non0lyctine powder-post beetles of Yunnan (China) with a new genus and new species (Coleoptera: Bostrichidae). Zootaxa 5091: 501–545. |
